# Small Ones to Fight a Big Problem—Intervention of Cancer Metastasis by Small Molecules

**DOI:** 10.3390/cancers12061454

**Published:** 2020-06-03

**Authors:** Dennis Kobelt, Mathias Dahlmann, Malti Dumbani, Nazli Güllü, Benedikt Kortüm, Miguel E. Alberto Vílchez, Ulrike Stein, Wolfgang Walther

**Affiliations:** 1 Department Translational Oncology of Solid Tumors, Experimental and Clinical Research Center, Charité University Medicine Berlin and Max-Delbrück-Center for Moelcular Medicine in the Helmholtz Association, Robert-Rössle-Straße 10, 13125 Berlin, Germany; dennis.kobelt@mdc-berlin.de (D.K.); mathias.dahlmann@mdc-berlin.de (M.D.); malti.dumbani@mdc-berlin.de (M.D.); nazli.guellue@mdc-berlin.de (N.G.); benedikt.kortuem@mdc-berlin.de (B.K.); miguel.alberto@mdc-berlin.de (M.E.A.V.); ustein@mdc-berlin.de (U.S.); 2 German Cancer Consortium (DKTK), Im Neuenheimer Feld 280, 69120 Heidelberg, Germany

**Keywords:** cancer metastasis, solid cancer, intervention, small molecules, inhibitors

## Abstract

Metastasis represents the most lethal attribute of cancer and critically limits successful therapies in many tumor entities. The clinical need is defined by the fact that all cancer patients, who have or who will develop distant metastasis, will experience shorter survival. Thus, the ultimate goal in cancer therapy is the restriction of solid cancer metastasis by novel molecularly targeted small molecule based therapies. Biomarkers identifying cancer patients at high risk for metastasis and simultaneously acting as key drivers for metastasis are extremely desired. Clinical interventions targeting these key molecules will result in high efficiency in metastasis intervention. In result of this, personalized tailored interventions for restriction and prevention of cancer progression and metastasis will improve patient survival. This review defines crucial biological steps of the metastatic cascade, such as cell dissemination, migration and invasion as well as the action of metastasis suppressors. Targeting these biological steps with tailored therapeutic strategies of intervention or even prevention of metastasis using a wide range of small molecules will be discussed.

## 1. Introduction

### 1.1. The Demanding Clinical Need for Metastasis Intervention

Despite the progress for treatment of solid cancers, metastasis remains the key issue impacting failure or success of cancer therapies. Metastatic dissemination of primary tumors is directly linked to patient survival. Metastasis is not an inherent property of all neoplastic cells [1]. Some cancers are highly aggressive forming metastases with high frequency, while others are rarely metastatic despite being locally invasive. But, metastasis is the most lethal attribute for cancer patients and counts for about 90% of all cancer deaths [2,3].

Further, metastatic spread critically limits successful therapies in many tumor entities [4]. The limited therapeutic success defines the clinical need for novel metastasis-inhibiting treatment strategies aiming at key events and drivers of metastasis formation by using small molecule drugs. We are focusing here on biomarkers acting as causal key drivers for metastasis, being involved in signaling pathways, promoting and driving the metastatic phenotype of cancer cells, which may serve as useful targets for small molecule-based restriction of metastasis formation.

### 1.2. Exploiting the Metastatic Cascade to Find Vulnerabilities for Metastasis Intervention

Here we dissect the metastatic cascade for novel approaches to combat metastasis formation, which arise upon reviewing the metastatic cascade [5,6]. The main steps of this cascade start with cellular transformation and tumor growth. This necessarily includes progressive growth of neoplastic cells and the availability of nutrients for the expanding tumor mass, initially supplied by simple diffusion. The second step is proliferation and angiogenesis. Here, the extensive vascularization must occur if a tumor mass is to exceed 1–2 mm in diameter. Angiogenic factors must be synthesized and secreted, thereby building a capillary network from the surrounding host tissue. The third step is detachment and invasion. Tumor cell detachment from the primary tumor mass is caused by loss of adhesion programs and invasion in the adjacent tissue is mainly characterized by degradation of the matrix using a variety of proteinases, both leading to increase in cell motility. This local invasion of the tumor cells into the host stroma paves the way of the detached and invasive tumor cell into circulation. The next step—intravasation, when tumor cells enter the blood vessel and circulation—is performed by single tumor cells or tumor cell aggregates. Although the majority of these circulating tumor cells are rapidly destroyed, some cells survive the circulation, staying dormant and are trapped in the capillary beds of distant organs. In the circulation, tumor cells interact with for example, platelets and lymphocytes. Then, circulating tumor cells arrest at distant organ sites by binding the endothelium of the vessels there [7]. During the extravasation step, educated tumor cells leave the circulation by rupture of the walls surrounding the vessel and penetration of the circulating tumor cells into adjacent tissue. The last step, completing metastasis formation, is the proliferation and the re-organization of the extracellular matrix (ECM) of the arrested tumor cells in the organs of the secondary site, essentially supported by an appropriate microenvironment. A newly generated vascular network of the micrometastases will help to evade destruction by host defenses. Metastases then grow into metastatic colonies, with about 50 cells will constitute a colony and continue to grow until macroscopic metastases are clinically detectable.

Thus, metastasis development is only possible when the “seed,” the tumor cells as the secondary site and the “soil,” the new surrounding organ, are compatible—the “seed and soil model” [8]. Further, since each of the steps of the metastatic cascade is dependent on clearly defined molecular pathways and networks, key targets of these signaling cascades can be identified and used for step-specific treatment [9]. Various interference opportunities have been developed using small molecules [10].

**Figure 1:**
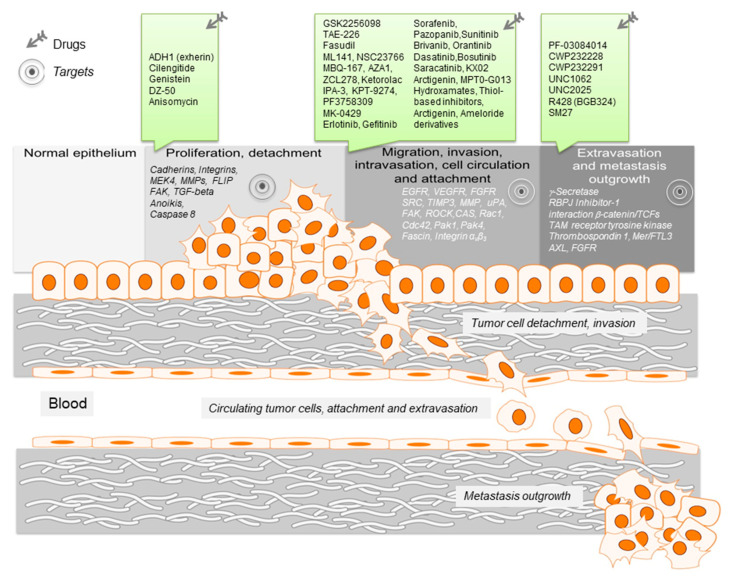
Schematic representation of the key events leading to metastasis. For the different metastatic steps important genes are listed, which represent drivers of the metastatic process. They enable cancer cell migration, invasion intra- and extravasation as well as metastasis outgrowth. Such genes represent promising targets for therapeutic interventions. In this regard, small molecule inhibitors are listed, which target particular steps in the metastatic process.

Here we will review single key steps of the metastatic cascade in the context of signaling pathways, key biomarkers thereof and targeting by small molecule drugs aiming specifically at these metastatic steps, which finally lead to metastasis restriction (Figure 1 and Table 1).

## 2. Targets for Therapeutic Intervention during Metastasis Formation

### 2.1. Tumor Cell Detachment—Principiis Obsta

Cell detachment from the primary tumor has been considered as initial step of metastasis [73]. The adhesion between cells but also to the ECM, is mediated through cell adhesion molecules (CAMs) [74]. Among them, calcium-dependent receptors such as cadherins and integrins play crucial roles. Their dysregulation causes the impairment of tissue integrity [74,75,76].

Cadherins are one group of the CAMs, which are crucial for proper cell-cell contact. Their dysregulation leads to loose cell-cell contacts allowing tumor cells to detach from the primary tumor and disseminate to a distant site [77,78]. For example, the expression of N-cadherin is elevated in many cancer cells and aberrant regulation of this molecule allows them to migrate and form metastases [79,80].

Integrins are further essential CAMs providing cell-ECM interactions [78,81]. While the extracellular domain of integrins binds to ECM molecules, the intracellular domain facilitates the attachment to the cytoskeleton via intracellular focal adhesions [82]. This binding not only regulates the cell adhesion but also provides the signal transduction between the cell and ECM via integrin activated signal molecules such as focal adhesion kinases (FAKs) and phosphatidylinositol 3-kinase (PI3K) [83]. The aberrant regulation of integrin increases cancer invasiveness via the dysregulation of these signal molecules. It also leads to the activation of matrix metalloproteinases (MMPs) responsible for ECM degradation [84,85]. MMPs are endopeptidases playing an essential role in physiological processes such as organogenesis, apoptosis and cell proliferation [86,87]. Their aberrant regulation leads to tissue damages, enables cancer cell motility and correspondingly causes spread of cell from primary tumors to distant sites [86,87,88].

Physiologically, when cells lost their cell-cell and ECM connection, an apoptotic process called anoikis is activated [89,90]. This process prevents survival and anchorage-independent growth of detached cells and thus hinders dissemination to distant sites. However, some cancer cells develop resistance mechanisms against this control mechanism.

The resistance to anoikis, together with changes in cell adhesion and cell polarity, is conjointly known as epithelial to mesenchymal transition (EMT). This process induces mesenchymal properties of cancer cells leading to increased motility and invasiveness.

These briefly described processes have prompted efforts to therapeutically target the detachment of cells from the primary tumors. They are focusing on three levels, to intervene in the cell detachment and survival of detached cells—(i) targeting CAMs, (ii) activating anoikis and (iii) breaking anoikis resistance.

CAMs represent one of the prominent targets to prevent metastasis initiation. ADH1 (exherin) inhibits the invasion and proliferation of some cancer types through binding and blocking the essential CAM component N-cadherin. It was tested in phase II clinical trials as monotherapy of different N-cadherin positive neoplasms. In clinical phase I settings the combination with cisplatin and gemcitabine for treatment of metastatic pancreatic or biliary tract cancer was tested, which in part led to stable disease [11].

Integrins are another prominent class of targets to prevent distant dissemination of tumor cells. The inhibition of integrin–ligand interaction not only decreases cellular growth but also induces apoptotic cell death. The integrin antagonist cilengitide, a cyclic pentapeptide, binds RGD (Arg-Gly-Asp)-dependent integrins, inhibits the ECM ligand-integrin interaction and thus induces apoptosis. The efficacy of this drug was tested in a clinical phase III trial for glioblastoma. In combination with the alkylating agent temozolomide and chemoradiotherapy the application of cilengitide however did not improve patient outcome [12]. By contrast, in a clinical phase I study cilengitide treatment showed an antitumor activity in combination with paclitaxel [13]. In a clinical phase II trial treating non-small lung cancer patients, cilengitide monotherapy was as effective as docetaxel [14].

Detachment of cancer cells from the ECM leads to conformational changes of integrin, followed by the transmission of outside-to-inside signals through pathways involving FAK. In particular cancer cells, which have high anoikis resistance show an elevated level of FAK expression [91]. Its inhibition by the isoflavanoid genistein reduced detachment of tumor cells and inhibited MMPs [15,16,17,18]. Genistein is tested in different clinical trials, including phase III [NCT00584532] clinical trial for prostate cancer.

Anoikis prevents survival of cells that lost their cell-matrix or cell-cell interactions [89,90]. Therefore, inducing anoikis by drug therapy is another promising approach to reduce the survival of detached cells. DZ-50 is a quinazoline-based compound that inhibits the epithelial and endothelial cell survival through inhibition of surface integrin β1. It reduces the tumor cell adhesion to the ECM by promoting anoikis and thus inhibits tumor growth in vivo [19].

One of the main pathways leading to the induction of anoikis is the death receptor pathway. This pathway is activated through binding of FAS Cell Surface Death Receptor (FAS) or TNF-Related Apoptosis-Inducing Ligand (TRAIL) to the extracellular domain of the death receptor. This leads to the activation of caspase 8, which cleaves downstream effectors, such as caspase 3 and 7 to finally induce cell death. Caspase 8 and FLICE inhibitory protein (FLIP) are structurally similar proteins. FLIP binds to the DISC complex and inhibits caspase 8 activation [92]. In malignant cells with metastatic potential, FLIP expression is increased, protecting cells from apoptosis. The antibiotic anisomycin (flagecidin) was identified as FLIP inhibitor in a compound library screen [20]. Thus, targeting FLIP with anisomycin leads to anoikis sensitization. The anoikis activating effect of this small molecule was not only shown in vitro for prostate cancer cells but its anti-metastatic effect was also corroborated in mouse studies [20,92]. Recently, a novel, first-in-class FLIP inhibitor was identified by molecular modelling. The respective lead compounds entered preclinical validation and characterization [93].

Cell detachment and survival of the detached cells are the initial steps of metastatic dissemination. Therefore, it is crucial to target these pathways to prevent dissemination of cells to distant sites.

### 2.2. Migration of Tumor Cells—Stop Moving

During malignant progression, tumor cells polarize towards chemoattractant gradients and engage in remodeling of the cytoskeleton to physically move away from the primary tumor [94]. This requires directed interaction with the ECM via transmembrane receptors (integrins, discoidin domain receptors) for ECM proteins (fibronectin, fibrinogen, collagen, etc.) [95,96]. Upon contact with ECM these receptors form focal adhesions, which in turn activate FAK with assistance by adapter proteins (talin, paxillin). FAK is an important hub of intracellular signaling and integrates integrin and growth hormone receptor signals to various target proteins. In cell migration, FAK orchestrates PI3K/AKT and Rho-GTPase signaling to exert polarized cell motility [97,98,99].

Rho, Rac and cell division cycle 42 (Cdc42) initiate and catalyze the polymerization of actin filaments during lamellipodia and filopodia formation [100,101]. RhoA recruits formin mDia, while Rac and Cdc42 recruit WASP proteins and the Arp2/3 complex [102,103]. The Rock proteins are effectors for Rho, while Cdc42 acts via myotonic dystrophy kinase-related Cdc42-binding kinase (MRCK) to foster actomyosin contractility for effective locomotion of the migrating cell [104]. Downstream of Rac/Cdc42, the family of p21-activated kinases (PAK) increases focal adhesion turnover and LIMK1-dependent actin depolymerization, resulting in cytoskeleton remodeling and migration [105,106].

Another druggable target involved in actomyosin remodeling, Fascin, ties up actin filaments during filopodia formation and crucially contributes to tumor cell motility in vitro and in vivo [107,108,109,110]. Fascin is a negative prognostic marker of cancer patient survival [111,112]. Certain members of small calcium-binding S100 proteins, most notably S100A4, have emerged as accomplice of cancer progression. S100A4 induces cell migration as a catalyst of interaction with F-actin and of myosin-IIa disassembly [113,114,115,116].

Due to its prominent position in cancer progression, FAK has been experimentally targeted with various inhibitors [117]. GSK2256098 intercepts the phosphorylation of FAK at tyrosine residue Y397 and delays pancreatic cancer cell wound closure in vitro [21]. This compound was recently tested clinically in glioblastoma [NCT01138033] and mesothelioma. It prolonged survival of patients with merlin-negative tumors [NCT01938443] [118].

The ATP-competitive FAK inhibitor TAE-226 also exerts efficacy against IGFR1 and was found to restrict glioma cell viability and motility in vitro [22]. Only recently, TAE-226 was able to prevent lung metastasis of orthotopically injected murine breast cancer cells in syngeneic mice [119].

Fasudil (HA1077) directly inhibits ROCK as an effector of RhoA and is already clinically approved in Japan for vasospasms due to its capability to suppress actin stress fiber formation and vascular muscle cell migration [23]. This finding was successfully recapitulated in cancer cells [120,121,122]. However, no clinical trial on cancer has considered fasudil so far.

Multiple novel Rho-GTPase inhibitors prevent pro-migratory cytoskeletal rearrangement and cell motility [123]. While RhoA inhibitors have not been promoted beyond biochemical analyses, Rac1 and Cdc42 inhibitors were promising in restricting cell migration in vitro [124]. Several molecules have been designed to compete with the nucleotide-binding pocket of Rac1 and Cdc42. EHT-1864 is particularly effective in preventing estrogen- and androgen-dependent Rac activation in breast and prostate cancer but might only serve as a prototype due to its adverse effect of platelet apoptosis in mice [25,125,126,127]. CID2950007 (ML141) and CID44216842 specifically target Cdc42 and restrict the motility of ovarian cancer cells [24].

Other compounds interfere with guanine nucleotide exchange factor (GEF) binding to Rac/Cdc42 directly. NSC23766 was designed to occupy Rac1’s binding pocket for the GEFs Trio and Tiam [26] and inhibited lamellipodia formation in lung cancer cells [128]. Its unacceptable toxicity, however, stimulated several optimizations leading to EHop-016, MBQ-167 and AZA1 [27,28,29,129].

Therefore, PAK1 as a major executor of Rac1-mediated migration, was targeted in an extensive in silico screen. Two compounds structurally unrelated to NSC23766 emerged as potent targeting pancreatic cancer cell migration while non-toxic towards normal pancreatic cells [130].

A variety of Cdc42 inhibitors have been established. ZCL278 intercepts activation of Cdc42 by Intersectin. It was effective in blocking actin-dependent migration of prostate cancer cells in vitro while having no effect on cell viability [32]. AZA197, derived from AZA1, occupies the nucleotide binding pocket of Cdc42 and prevented colon cancer cell motility and xenograft implantation in vivo [30].

Despite promising effects in preclinical studies, none of the small molecules discussed have successfully advanced to testing in humans [124,130]. Nevertheless, the R-enantiomer of the common NSAID ketorolac was repurposed in ovarian cancer cells to decrease Cdc42 dependent filopodia formation and cell migration. In a clinical phase III trial the drug was tested for high risk breast cancer patient treatment [31].

Extensive effort has also been put in the deployment of PAK inhibitors, particularly targeting PAK1 and PAK4 [131]. IPA-3, the only isoform-selective, allosteric inhibitor of PAK1, stabilizes PAK1 in its autoinhibitory state. Thereby, PAK1-dependent cell membrane ruffling is blocked [33,132]. IPA-3 prevents PAK1 dependent recruitment of WAVE2 and lamellipodia formation. This results in reduced migration and metastasis of i.v.-injected esophageal cancer cells [133,134]. KPT-9274 and KPT-8752 can reduce PAK4 expression, resulting in inhibition of growth and migration of renal cancer cells [34]. A clinical study gauging the safety of KPT-9274 is currently recruiting (NCT02702492). Another PAK4-inhibitor, PF3758309, was shown to restrict lung cancer cell motility. It was prematurely terminated in a clinical trial due to intolerable adverse effects. This indicates the need for further optimization of this compound (NCT00932126) [35,36].

Treatments with fascin inhibitors NP-G2-029 and NP-G2-044, which prevent interaction with actin filaments, resulted in reduced migration, invasion and metastasis of breast cancer cells [135,136]. NP-G2-044 is currently in a phase I trial in patients with metastatic disease [NCT03199586].

Integrins as mediators of cell migration also support tumor cells in circulation (CTCs) and metastatic settlement in distant organs. Integrin α_v_β_3_ is instrumental for the extravasation of breast cancer cells. Tumor cells recruit platelets within the capillary system of the metastatic site, which in turn release a wide variety of tissue-remodeling factors and facilitate trans-endothelial migration [137]. MK-0429, an orally bioavailable small molecule, prevents lung metastasis of i.v. injected melanoma cells and is a major contestant against mainly antibody-based integrin inhibition [37,138].

Despite improvements in surgical techniques, that seem to obviate any use of migration inhibition of primary tumors, the consideration of the molecules listed above could have merit in the treatment of unresectable malignancies or in for example, neo-adjuvant settings.

### 2.3. Invasion Intervention—Stop the Invaders

Invasion through ECM, intravasation to vasculature and extravasation at the distant site of tumor cells is regulated by complex signaling. It involves formation of invadopodia, secretion of proteases as well as factors that attract tumor cells to the metastatic site. In this context, factors that constitute the metastatic niche and the proper environment are essential to promote tumor cell invasion [78,139].

An important event in tumor cell invasion is the formation of F-actin-rich invadopodia as membrane protrusions. This ensures cellular movement and invasion through the ECM [78,140,141,142]. Invadopodia are important to clear the tumor cell path by degradation of cell-cell junctions and of the ECM. Formation of invadopodia is triggered by growth factors such as EGF, PDGF, basic FGF and also by integrins [78]. These extracellular stimuli activate PI3K and Src signaling leading to actin re-modeling. This is essential to provide the mechanical forces of cell movements [140,141]. Particularly Src signaling has been identified as key event in this process. This is further supported by adhesion domains, which bind to the ECM and provide the anchor promoting directed movement. Further, activity of invadopodia is associated with the action of MMPs and of serine proteases for effective invasion [143,144]. Serine proteases such as uPA not only degrade the ECM but are also known to proteolytically activate growth factors, for example, HGF, TGF-alpha or basic FGF [78].

These briefly introduced processes of tumor cell invasion are targets for therapeutic intervention. Considering the sequence of events impacting tumor cell invasion, three levels are useful for invasion intervention, which also affect invadopodia formation—(i) stimulation by growth factors, (ii) invasion-promoting signaling and (iii) protease activation.

Growth factors play a decisive role in inducing invasive properties of tumor cells [145,146,147]. Growth factor receptors, such as EGFR, PDGFR, basic FGFR and VEGFR are in focus. Apart from antibody-based interventions, small molecule inhibitors are in clinical use or under development. For EGFR-signaling erlotinib and gefitinib are known tyrosine kinase inhibitors, whereas sorafenib, sunitinib and pazopanib are inhibitors of the VEGF receptor [38,39,40,41]. Further, FGFR and also the VEGFR function is antagonized by brivanib (BMS-582664), a prodrug, which is converted to the ATP-competitor BMS-450215 [42]. PDGFR autophosphorylation and therefore receptor activation can be inhibited by the adenine mimetic drug orantinib (SU6668), which also acts on FGF-1, due to structural similarities of the ATP binding sites of the two receptors as target motive for the drug [43]. For all these, mostly multitarget inhibitors, anti-metastatic and anti-invasion activity has been demonstrated.

As mentioned, Src and Src-signaling play an important role in invasion. Its inhibition was shown to intervene in invasion and metastasis. Small molecule inhibitors, which interfere with Src activity and signaling, are the clinically used drugs dasatinib and bosutinib (SKI-606), the dual kinase inhibitor saracatinib (AZD-0530) and the dual Src/tubulin inhibitors KX02, KX2-391 [44]. Among those, dasatinib has been shown to inhibit tumor growth and metastasis formation of an orthotopic prostate cancer model [45,46]. This was associated with drug-mediated reduction in cancer cell migration and invasion. Saracatinib inhibits the invadopodia regulatory proteins FAK, p130 Crk-associated substrate (CAS) and contactin in HNSCC [47]. Such small molecule inhibitors of pathways, which are essential for invadopodia formation and function, indicate the effectiveness of intervention strategies at this driving step of cancer metastasis.

The main function of invadopodia for cancer cells is promotion of matrix degradation to support cancer cell invasion. In this regard targeting proteases is an additional level for effective intervention and prevention of metastasis. In this context, MMPs (e.g., MMP-2, MMP-9) are valuable targets. MMPs are members of zinc-endopeptidases with proteolytic activity against a broad spectrum of ECM substrates to support tumor cell invasion [148,149]. Tumor cells express MMPs at their leading edges to degrade collagen fibers to open the invasion path. Based on this, inhibition of invasion by MMP interference might contribute to inhibition of the entire metastatic process [48,49,50]. For such an approach, numerous small molecule inhibitors have been developed. They belong to the group of hydroxamates (batimastat, marimastat, prinomastat, solimastat etc.), thiol-based MMP inhibitors (e.g., rebimastat, tanomastat) and other MMP inhibitors, such as carbamoylphosphonate cis-ACCP, pyrimidine-trione-like Ro28-2653 or the sulfonamide derivative S-3304. Clinical testing of such inhibitors however revealed low efficacy of MMP intervention. This is potentially due to low selectivity of the drugs or due to emergence of resistance mechanisms in treated tumors. Another resistance mechanism is the switch of tumor cells from protease-dependent to protease-independent invasion to circumvent the inhibitory activity of applied small molecule drugs. This is the rational, by which the tissue inhibitors of metalloproteases (TIMPs) came into focus. Here, small molecules are of increasing interest, which stimulate TIMP expression to inhibit the malicious action of MMPs in tumor invasion and metastasis [51]. Such activity has been tested for compounds like the organo-sulfur compound diallyl-disulfide, the lignan arctigenin or the arylsulfonamide derivative MPT0G013 and many others, which all increased TIMP-3 expression and inhibited tumor cell migration and invasion.

Due to the migration, invasion and metastasis promoting function of the serine protease uPA, numerous approaches are aiming at inhibition of its proteolytic activity [150]. Small molecule inhibitors, such as the ameloride derivatives B-428 and B-623 were shown to inhibit prostate and also breast cancer growth and metastasis in vivo [52]. Similarly, encouraging results have been obtained by testing WX-671 and its prodrug WX-UK1 in clinical trials for treatment of solid tumors [53,54]. Such uPA targeting approaches indicate the therapeutic value for tumor suppression and metastasis reduction.

In summary, there is a plethora of small molecules available, which act on processes of invadopodia action, cell migration and invasion. Their use for intervention in signaling processes of migration and invasion and combination with other drugs that interfere in other essential steps of metastasis formation will contribute to improved anti-metastatic therapy of cancer.

### 2.4. Metastasis Outgrowth—Intervention of Settlement

Tumor cells, which enter the circulation must survive various challenges before they can form clinically manifest metastases. A small percentage of cancer cells will survive after leaving the primary tumor and infiltrate distant organs. These tumor cells can reside in niches and remain dormant for years until receiving cues to proliferate [151,152,153,154]. As these cells are in a dormant state, it is assumed that they are unaffected by conventional chemotherapy targeting actively dividing cells [155]. Studies have shown that cell’s fate to remain dormant or proliferate depends on the cues from the tumor microenvironment, for example, Notch, Wnt, p38 signaling pathways or growth factor effects.

Notch signaling, pivotal for cell-cell communication, is often deregulated and mutated in cancer. Following ligand binding, the Notch receptor is cleaved by proteolytic activities of ADAM10 and γ-secretase [156,157]. This releases the Notch extra- and intracellular domains (NECD, NICD). Cleaved NICD translocates to the nucleus and forms complexes with the recombination signal binding protein for immunoglobulin kappa J region (RBPJ, former CSL) and mastermind-like (MAML). This complex induces transcription of Notch target genes. The C-terminus of NICD contains a PEST domain targeting NICD for rapid degradation. The PEST domain is often mutated in cancer, thus impairing the degradation of Notch [158]. Activation of Notch promotes STAT3 phosphorylation resulting in activation of cancer stemness pathways [159]. Various strategies to intervene in Notch signaling (cleaving enzymes, nuclear translocation and intracellular complex formation of NICD) have been explored [158,160,161]. PF-03084014 (PF-4014/nirogacestat) is a potent γ-secretase inhibitor hindering STAT3-Notch1 interaction [159]. It acts by inhibiting the Ser/Thr phosphorylation of STAT3, crucial for Notch1 interaction. This reduces survival signals mediated by for example, MEK/ERK and PI3K pathways. Due to activation of BAK, BAX and Caspase 3 apoptosis is increased [55]. When used in combination with glucocorticoids to treat T-cell acute lymphoblastic leukemia (T-ALL) in in vivo models, the anti-tumor response is enhanced compared to monotherapy [56,57]. Interestingly, PDX models with mutations in the PEST domain of Notch were also sensitive to PF-03084014 [58]. Currently, PF-03084014 is in a phase III randomized double-blind clinical trial for treatment of aggressive fibromatosis/desmoid tumors (NCT03785964) [59]. Another targeted small molecule inhibitor intervening in Notch pathway is RBPJ Inhibitor-1 (RIN1). RIN1 is an inhibitor disrupting the interaction between NICD and its transcriptional effector, RBPJ. Targeting with RIN1 allows to selectively modulate Notch signaling avoiding off-target effects observed for other inhibitors targeting this pathway [162].

In addition to the signaling pathways involved in proliferation as exemplified above, the niches harboring dormant cells also serve as attractive targets. Dormant cells reside in niches that are highly similar to hematopoietic stem cell (HSC) niches [163]. Signals like CXCL4/CXCL12 or TGF-beta that prompt the production of stem cells, also activate dormant cells. One such example is granulocyte-colony stimulating factor (G-CSF), a glycoprotein that is commonly supplemented with chemotherapy to stimulate the release of stem cells from bone marrow, also releases dormant cancer stem cells [164,165]. Aberrant activation of Wnt/ β-catenin pathway is usually observed in metastatic tumors [166]. CWP232228 is a highly potent small molecule inhibitor identified in a high-throughput screen. CWP232228 prevents binding of β-catenin to TCF proteins in the nucleus thereby reducing tumor formation and metastasis. In vivo CWP232228 suppressed tumor growth in colon cancer models [60]. CWP232291, closely related to CWP232228, completed a phase I trial. This trial resulted in defining the safe and tolerable dose of CWP232291 alone and in combination therapy for phase II studies. Currently, CWP232291 is in a phase IIa clinical trial for leukemia (NCT03055286) [61]. IC2, another derivative of CWP232228, has demonstrated effective suppression of liver cancer stem cells and cancer stem cell marker positive population in vitro and in vivo [167]. Future research developing small molecules capable to modulate stem cells and hematopoietic cell lineage, may also eliminate cancer stem cells and serve as promising therapeutic approach.

Another key molecule involved in the regulation of hematopoietic stem cells is growth arrest specific 6 (Gas6) [168]. Gas6 is a ligand for TRYO3, AXL and MER (TAM) family of receptor tyrosine kinases [169,170]. Studies have indicated that GAS6 is involved in cell invasion and migration through the GAS6/AXL axis. Importantly, Gas6 is involved in dissemination of tumor cells to the bone marrow. There, these cells enter cell arrest and survive for long periods undetected [171,172]. As tyrosine kinase receptors are structurally and functionally similar to a certain extent, it is challenging to develop selective inhibitors [173]. Nevertheless, with the recent advances selective inhibitors that target Gas6 receptors such as UNC1062 (Mer) and UNC2025 (Mer/FTL3) have been developed [62,63]. R428 (BGB324) is the first Axl inhibitor that is in phase II clinical trial for multiple cancers (NCT02488408, NCT03824080) [64]. In summary, these novel selective compounds not only establish the integral role of GAS6 and TAM receptors in regulating tumor dormancy but also serve as tools for further development of selective TAM inhibitors.

Angiogenesis is a fundamental process not only during tumor growth but also for organ colonization [174]. VEGF and FGF are key pro-angiogenic growth factors, known to induce angiogenesis and cell proliferation [175,176,177]. Thrombospondin 1 (TSP1), a protein belonging to the family of ECM proteins, plays a key role in regulating angiogenesis. TSP1 is capable of inhibiting angiogenesis by binding and sequestering FGF2 and thus prevents its activity. By exploring the FGF2 binding sequences of TSP1 novel anti-angiogenic molecules are designed [65]. One such example is SM27 (naphtalenosulfonic acid derivative) developed by Colombo et al. SM27 inhibits the interaction of FGF2 with its receptors FGFR1 and HSPGs thus hindering the formation of ternary complex required for FGF2 signal transduction [65,66].

In summary, there is a need to improve our understanding of the signaling pathways that regulate cancer cell dormancy and the niches homing the dormant tumor cells in order to develop small molecule inhibitors to successfully eliminate seeds of future metastasis.

### 2.5. Metastasis Suppressors—Natural Borne Inhibitors

Instead inhibiting pro-metastatic factors, small molecules are also applied to induce factors inhibiting steps in the metastatic process, the metastasis-suppressor genes (MSG). A literature-based resource summarizes 194 experimentally verified MSGs, including functional annotations, gene expression and methylation data [178].

MSGs act in several cellular pathways linked to metastasis, for example, signaling pathways, apoptosis, modulation of the cytoskeleton and altered cell adhesion, comprehensively reviewed in Reference [179]. The first identified metastasis suppressor in mouse models for metastasis formation was nucleoside diphosphate kinase Nm23 [180,181,182]. Nm23 represses metastasis formation in part by phosphorylating kinase suppressor of Ras (KSR), leading to decreased ERK activation [183]. Over time, more MSGs were identified which affect MAPK signaling, like Ras kinase inhibitory protein (RKIP) and MAPK kinases 4, 6 and 7 [184,185,186,187]. Other signaling-related MSGs include the secreted G-protein-coupled receptor (GPR) ligand precursor KiSS-1 and Src-suppressed C kinase substrate (SSeCKS), acting as a scaffold for signaling factors [188,189].

MSGs are also involved in cell adhesion and cytoskeleton regulation, leading to reduced migration. E-cadherin, CD44 and CD82 are transmembrane glycoproteins. These proteins mediate cell-cell interactions and modulate plasma membrane receptor activity or interaction of membrane proteins with the cytoskeleton, respectively [190,191,192]. Rho-GTPase-activating protein 7, deleted in liver cancer (DLC)-1 and Rho-GDP-dissociation inhibitor alpha (Rho-GDIα) are more directly involved in cytoskeleton regulation [193,194,195]. But also repressors of gene transcription have been identified as MSGs, like breast cancer metastasis suppressor (BRMS) 1, associated with histone deacetylation (HDAC) complexes and surprisingly the proto-oncogene MYC [196,197,198].

Clinically, MSG expression resulted in favorable prognosis for the majority of solid cancers [199,200]. Less clear is the value of MSGs as predictive biomarkers. Preclinical data link Nm23 expression with increased sensitivity to cisplatin treatment [201]. A retrospective clinical analysis reports better chemotherapy treatment response in Nm23-expressing tumors [202]. A recent study links a higher promoter methylation status of the metastasis suppressor NK6 homeobox 1 (NKX6.1) resulting in reduced expression, with a less favorable prognosis of stage II CRC patients after receiving adjuvant chemotherapy [203]. This points to a promising strategy to re-express MSGs in cancer cells. Focusing on epigenetic regulation mechanisms in metastasis formation, inhibitors of DNA-methylation, histone-methylation or -deacetylation are reported to increase MSG expression [204].

Screening for cancer-related genes downregulated by DNA-methylation in gastric cancer identified the induced expression of KiSS-1 along with its receptor GPR54 upon treatment with 5-aza-2’-deoxycytidine/decitabine [67]. The induction of KiSS-1/GPR54 upon DNA-demethylation was confirmed in endometrial cancer and might serve as therapeutic strategy to inhibit metastasis formation in additional cancers [205]. Methylated CpG-islands were also found in the promoter region of Nm23. Treatment with decitabine increased Nm23 expression but also BRMS1 or RKIP in breast cancer cells, resulting in reduced cell migration and invasion [70,206,207].

Histone methylation can silence gene expression in cancer cells, mediated by histone methyltransferases (HMT) like enhancer of zeste homolog (EZH) 2. EZH2 is frequently upregulated in cancer. Its inhibition by 3-deazaneplanocin A (DZNep) reduces metastasis formation by re-expression of E-cadherin and DLC-1 [68,208]. EZH2 is also involved in transcriptional repression of RKIP in breast and prostate cancer, supporting a potential anti-metastatic treatment by targeting HMTs [209,210].

Similarly, expression of silenced MSGs can be restored by inhibition of histone deacetylases (HDACs) using drugs like suberanilohydroxamic acid (SAHA). SAHA treatment re-induced the expression of E-cadherin, decreasing EMT and cell motility [69,211]. HDAC inhibition by SAHA also increased the expression of RKIP, SSeCKS and Nm23 in various cancer types [70,212,213,214]. Thus, upregulating MSG expression by remodeling the epigenetic landscape in cancer cells represents a promising anti-metastatic treatment strategy for high-risk patients, either in mono- or combination therapies [215,216,217].

MSGs can also be induced by non-steroidal anti-inflammatory drugs (NSAIDs). Generally, pre- or post-diagnostic use of NSAIDS correlates with reduced distant metastasis [72,218]. Their anti-metastatic effect was identified by the upregulation of MSGs like Nm23 in different cancer cells [219,220,221]. Similarly, COX-2 inhibition by NSAIDs upregulates E-cadherin, partially by decreasing its promoter methylation [222,223,224].

Many MSG promoters are reported to be repressed through NF-κB binding sites. This mechanism is supported by the upregulation of RKIP expression in breast cancer cells upon inhibition of NF-κB signaling by dehydroxymethylepoxyquinomicin in breast cancer [70]. Also CD82-expression is regulated by active NF-κB signaling but its repression or induction strongly depends on the cell type or at least the mutation status of p53 [225,226]. NF-κB signaling also decreases Rho-GDIα on protein level by upregulating its E3 ubiquitin ligase, triggering proteasomal degradation of ubiquitinylated Rho-GDIα [227].

Targeting NF-κB signaling is promising to therapeutically inhibit metastasis formation. It was proven to decrease the metastatic potential of different cancer cells [228,229,230]. Specific inhibitors of NF-κB signaling in solid cancers are currently tested in animal models (reviewed in Reference [231]). Interestingly, BRMS1 and KiSS-1 can decrease NF-κB-mediated target gene expression and thus suppress cancer cell motility and invasiveness [232,233,234,235,236,237].

Re-activation of MSG expression can be achieved by repositioning drugs from various classes (summarized in Reference [223]). Preclinical reports link the application of individual drugs to a decrease in cancer progression or metastasis formation but only little literature is available for clinical trials with focus on metastasis-suppressors. The promising preclinical strategy of upregulating Nm23 expression in breast cancer by progestines was evaluated in a prospective clinical phase II trial but with only limited success [71,238].

The main obstacle in the translation of MSG-expression by small molecules is the range of affected cellular mechanisms, raising the danger to induce severe side effects. This rather discourages the strategy of systemic MSG-induction in anti-cancer therapy. But the emerging concept of personalized/precision medicine, combined with deeper insight into molecular mechanisms of metastasis formation, might circumvent this issue.

## 3. Conclusion and Future Prospects of Anti-Metastatic Therapy

Cancer cell migration, invasion and metastasis belong to the hallmarks of cancer, which severely limit therapeutic options and in result of this, also limit patient survival. For example, colorectal cancer patients have a good prognosis if diagnosed before metastases have formed. If metastases are already present, long-term survival is reduced and therapeutic options are restricted by the systemic spread of the disease. This situation has not substantially changed for metastasized patients in the past decades. Therefore, profound knowledge of processes leading to metastasis formation is of crucial importance. If we understand, how and when cancer cells migrate, invade, circulate and colonize distant sites, the successful treatment for long-term survival of patients becomes a realistic option. To reach this goal, patients at high risk need to be identified by strong biomarkers, which are in the best scenario also causative molecules for metastasis formation. For these patients, tailored treatments targeting cells at specific steps in the metastatic cascade will allow to disrupt the aberrantly regulated processes for efficiently inhibiting the metastasis formation and progression. Use of specific inhibitors will interfere in the best scenario in early events of the metastatic process, hindering metastasis formation as early as possible. Since cancer cells activate different mechanisms for effective migration, invasion, apoptosis prevention and so forth, the combination of small molecule drugs targeting such different signaling pathways of the metastatic process will assure improved treatment of metastatic cancer. The knowledge on how such pathways complement or replace each other to ensure cancer cell metastasis will be the basis for more effective combination therapies using small molecules. For this, research has to find inhibitors, which specifically interfere with metastasis drivers. In addition, search for novel and powerful regulators of metastasis, knowledge of aberrantly regulated signaling pathways and use of new or repositioned drugs for these targets opens new therapeutic options.

## Figures and Tables

**Table 1:** The table summarizes the small molecule drugs and their targets discussed in this review.

Biologic Process, Pathway or Biological Function	Direct Target	Drug	Ref.
**Proliferation and detachment**			
Cadherin signaling	N-cadherin	ADH1 (exherin)	[11]
Integrin signaling	α_v_β_3_ and α_v_β_5_ integrin	Cilengitide	[12,13,14]
FAK/TGF-beta signaling, MMPs	MEK4	Genistein	[15,16,17,18]
Integrin signaling, anoikis	Integrin β1	DZ-50	[19]
Anoikis, caspase 8 activity	FLIP	Anisomycin	[20]
**Migration, invasion, intravasation, circulation and attachment**			
Focal Adhesions, cytoskeletal remodeling	FAK	GSK2256098, TAE-226	[21,22]
Stress fiber formation	ROCK	Fasudil	[23]
Actomyosin contraction	Cdc42	ML-141/CID2950007, CID44216842	[24]
Actomyosin contraction	Rac, Cdc42	EHT-1864, NSC23766, Ehop-016, MBQ-167, AZA1, AZA197, Ketorolac	[25,26,27,28,29,30,31]
Actomyosin contraction	Rho	ZCL278	[32]
cytoskeleton turnover	PAK	IPA3, KPT-9274, PF3758309	[33,34,35]
extravasation of circulating tumor cells	α_v_β_3_ integrin, fascin	MK-0429, NP-G2-029, NP-G2-044	[35,36,37]
EGFR mediated signaling	EGFR	Erlotinib, Gefitinib	[38,39,40]
VEGFR signaling	VEGFR	Sorafenib, Pazopanib, Sunitinib	[41]
FGFR and VEGFR signaling; ATP competitor	FGFR, VEGFR	Brivanib	[42]
PDGFR and FGFR1 signaling; ATP competitor	FGFR	Orantinib	[43]
SRC signaling	SRC	Dasatinib, Bosutinib	[44,45,46]
SRC signaling, invadopodia formation	FAK, CAS	Saracatinib	[44,47]
SRC signaling, tubulin polymerization	SRC/tubulin	KX02, KX2-391	[44]
MMP activity, invasion	MMP	Hydroxamates, thiol-based inhibitors, S3304, cis-ACCP, Ro-28-2653, B-428, WX-671	[48,49,50]
TIMP expression for MMP interference	TIMP3	Arctigenin, diallyl-disulfide, MPT0-G013	[51]
uPA mediated growth factor activation	uPA	ameloride derivatives	[52,53,54]
**Extravasation and metastasis outgrowth**			
Notch signaling	γ-secretase	PF-03084014	[55,56,57,58,59]
Wnt signaling; transcriptional activity of β-catenin	β-catenin	CWP232228	[60]
Wnt signaling; downstream apoptotic cell death pathway	β-catenin	CWP232291	[61]
TAM signaling; oncogenesis and anti-apoptotic activity	Mer	UNC1062	[62]
TAM signaling; prosurvival and anti-apoptotic pathways	Mer/FTL3	UNC2025	[63]
TAM signaling; prosurvival, proinflammatory, EMT pathways	AXL	R428 (BGB324)	[64]
Angiogenesis; FGF signaling	FGF2-FGFRR1-HSPGs	SM27	[65,66]
**Metastasis Suppressors**			
epigenetic regulation of gene expression: DNA methylation	DNA methyltransferases	5-aza-2’-deoxycytidine (decitabine)	[67]
epigenetic regulation of gene expression: histone methylation	EZH2	3-deazaneplanocin A (DZNep)	[68]
epigenetic regulation of gene expression: histone acetylation	histone deacetylases	suberanilohydroxamic acid (SAHA)	[69]
NFκB signaling pathway	NFκB	dehydroxymethylepoxyquinomicin	[70]
steroid hormone signal transduction	progesterone receptor	aedroxyprogesterone acetate (MPA)	[71]
prostaglandin signal transduction	Cox-2	non-steroidal anti-inflammatory drugs (NSAIDs)	[72]
